# Supplementary material for: One-Step Synthesis of Nanoliposomal Copper Diethyldithiocarbamate and Its Assessment for Cancer Therapy
Source: Pharmaceutics. 2022 Mar 14;14(3):640. doi: 10.3390/pharmaceutics14030640 (PMC8952320; doi:10.3390/pharmaceutics14030640)
Supplement: Supplementary file 1 [file pharmaceutics-14-00640-s001.zip › pharmaceutics-1626661-supplementary.pdf]

pharmaceutics

Article

Supplementary Material: One-Step Synthesis of Nanoliposomal Copper Diethyldithiocarbamate and its Assessment for Cancer Therapy

Radu A. Paun, Daciana C. Dumut, Amanda Centorame, Thusanth Thuraisingam, Marian Hajduch, Martin Mistrik, Petr Dzubak, Juan B. De Sanctis, Danuta Radzioch and Maryam Tabrizian

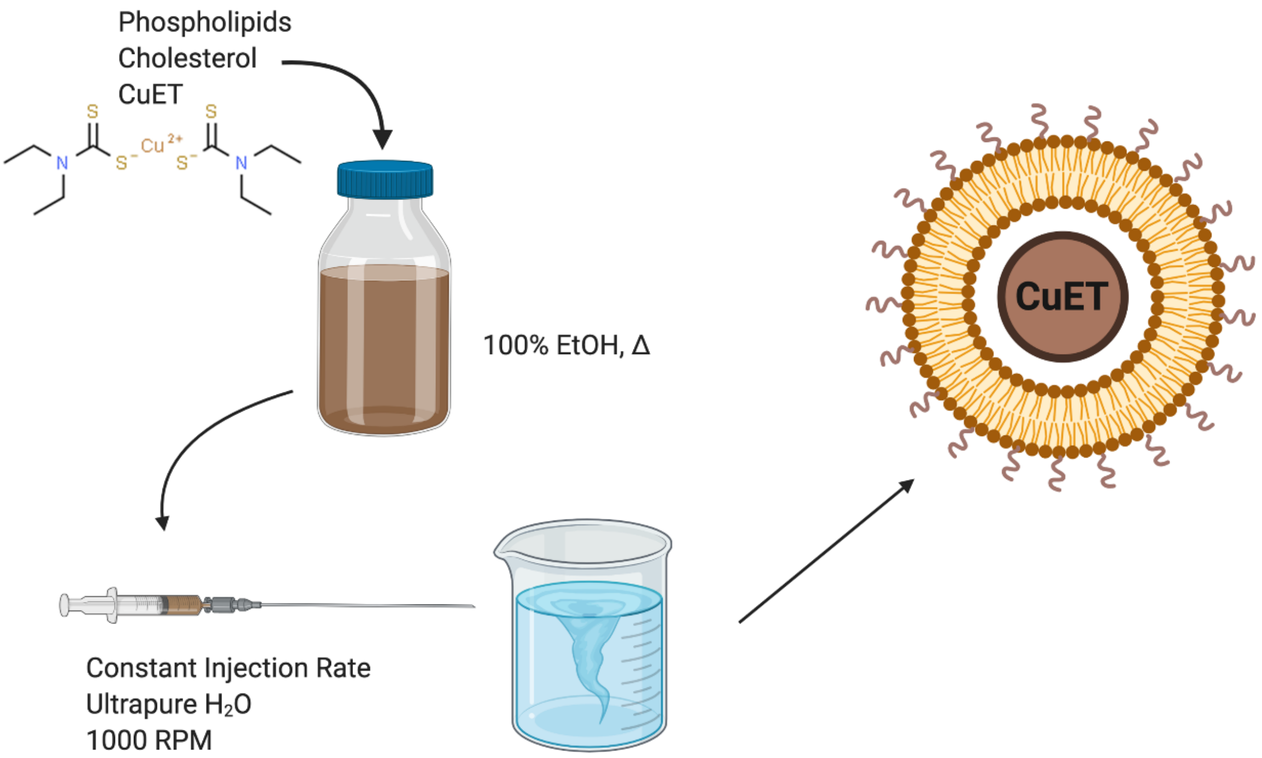

Figure S1. Schematic representation of LP-CuET synthesis using the modified ethanol injection method. CuET, phospholipids, and cholesterol are diluted in absolute ethanol and heated to 50 °C. The hot solution is then injected into rapidly stirring water to obtain LP-CuET.

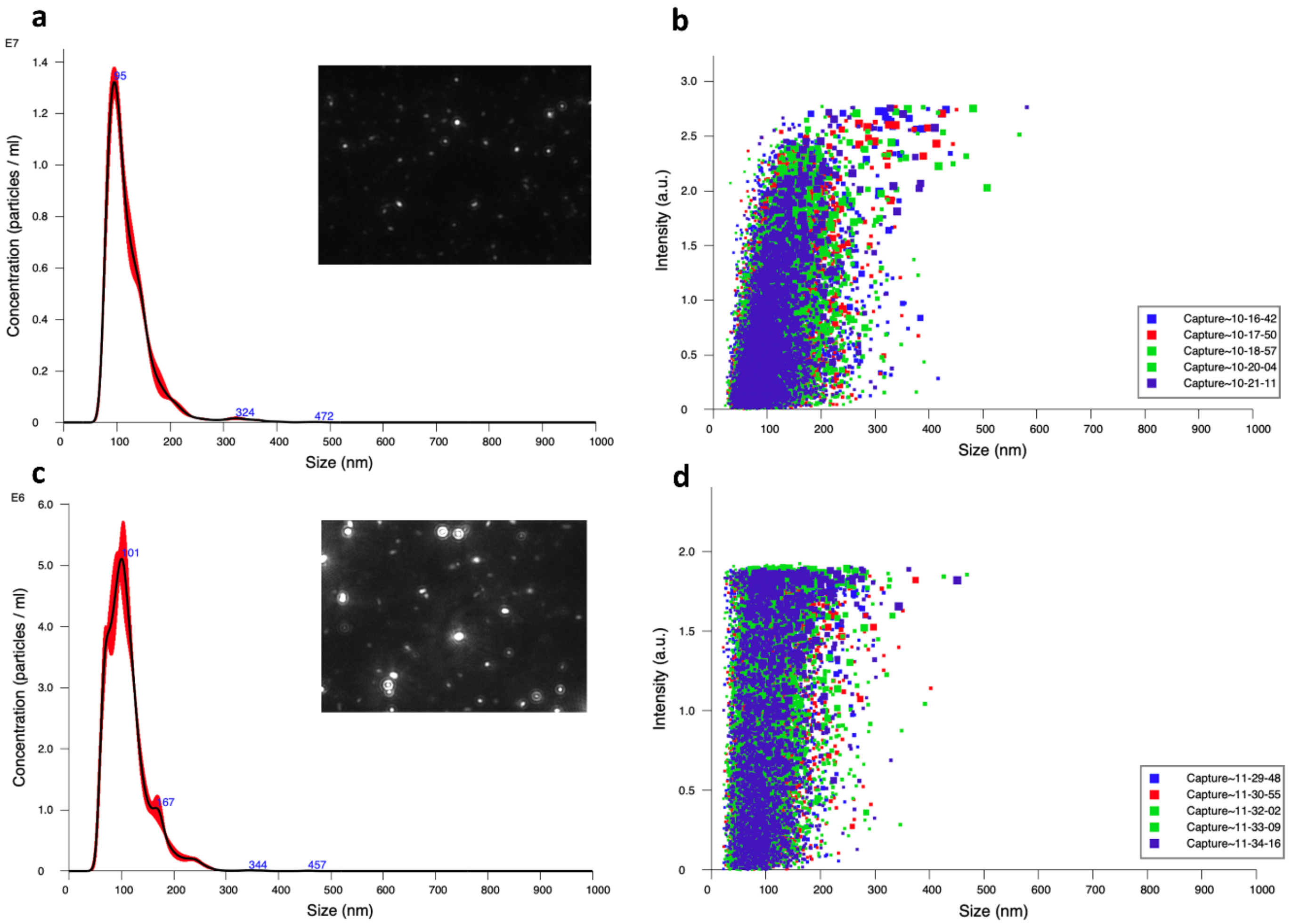

Figure S2. Concentration and intensity scatter plots of **(a–b)** LP-Control and **(c–d)** LP-CuET showing monodisperse distributions of the liposomes and their concentrations. E6, E7 = 106, 107. LP-CuET nanoparticles exhibited a brighter signal (insert) as compared to control liposomes.

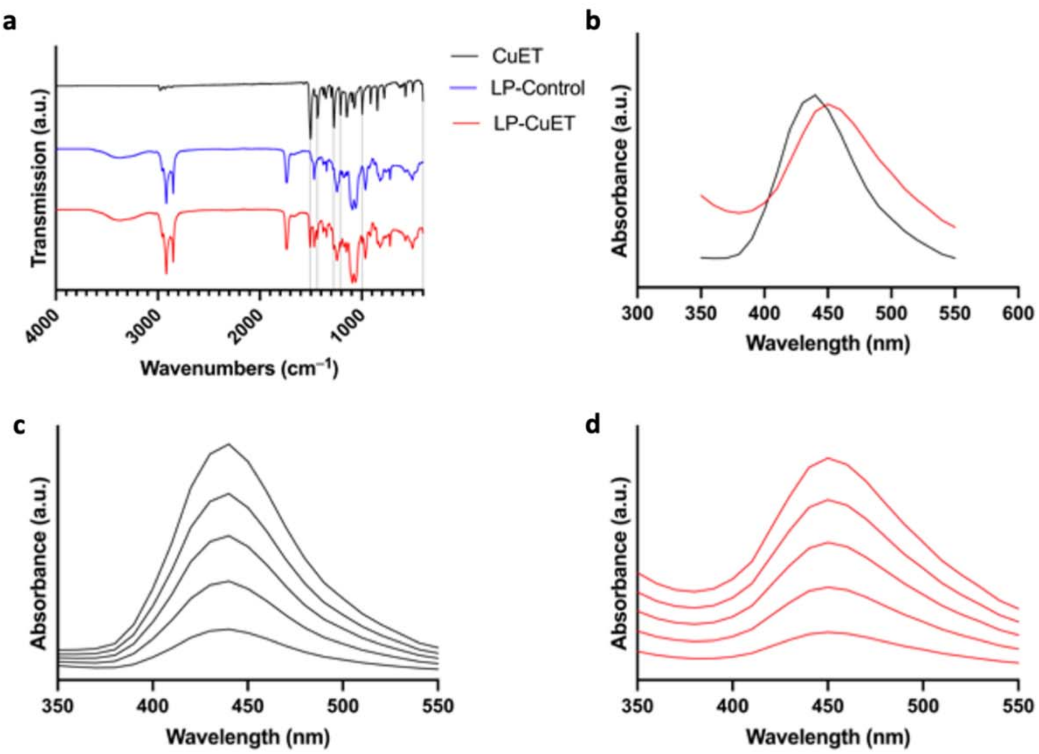

Figure S3. (**a**) Zoomed out FTIR graph of CuET, LP-Control, and LP-CuET. **(b)** Overlapping absorbance spectra of CuET in DMSO and LP-CuET in water, 450 nm was used for extrapolation measurements. **(c,d)** Concentration-dependent absorption spectra of CuET in DMSO and LP-CuET in water used for the standard curves.

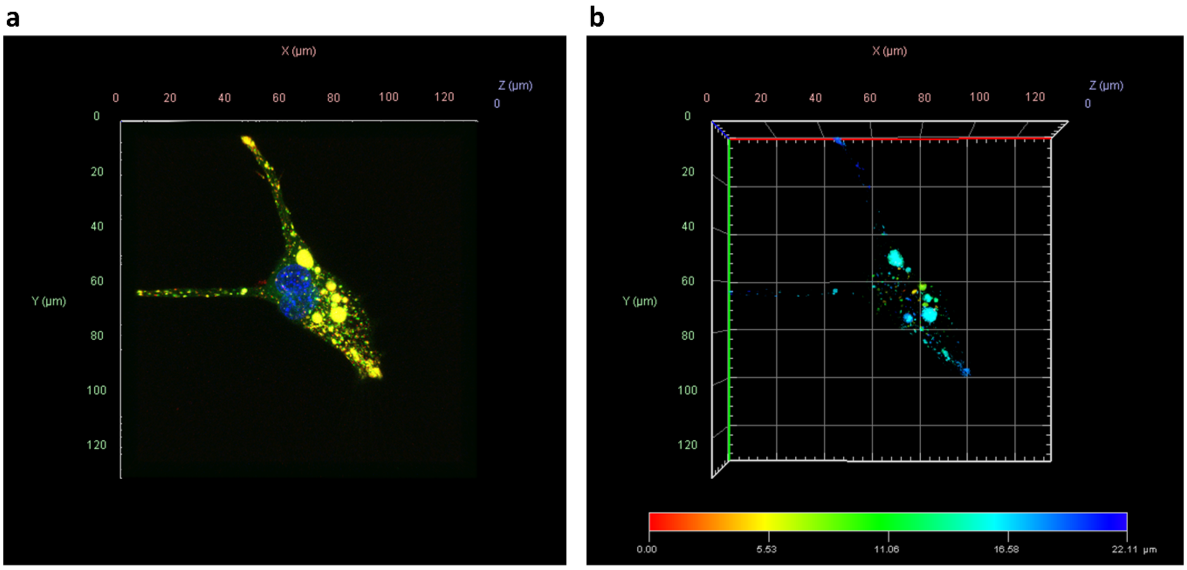

Figure S4. (a) two-dimensional and **(b)** depth plot of confocal images of live YUMM 1.7 cells treated with fluorescent LP-Control (red) and the acidic vesicle stain cytopainter (green) for 6h. Images are showing the spatial cellular distribution of drug-containing endocytic vesicles, including larger agglomerations around the nucleus.

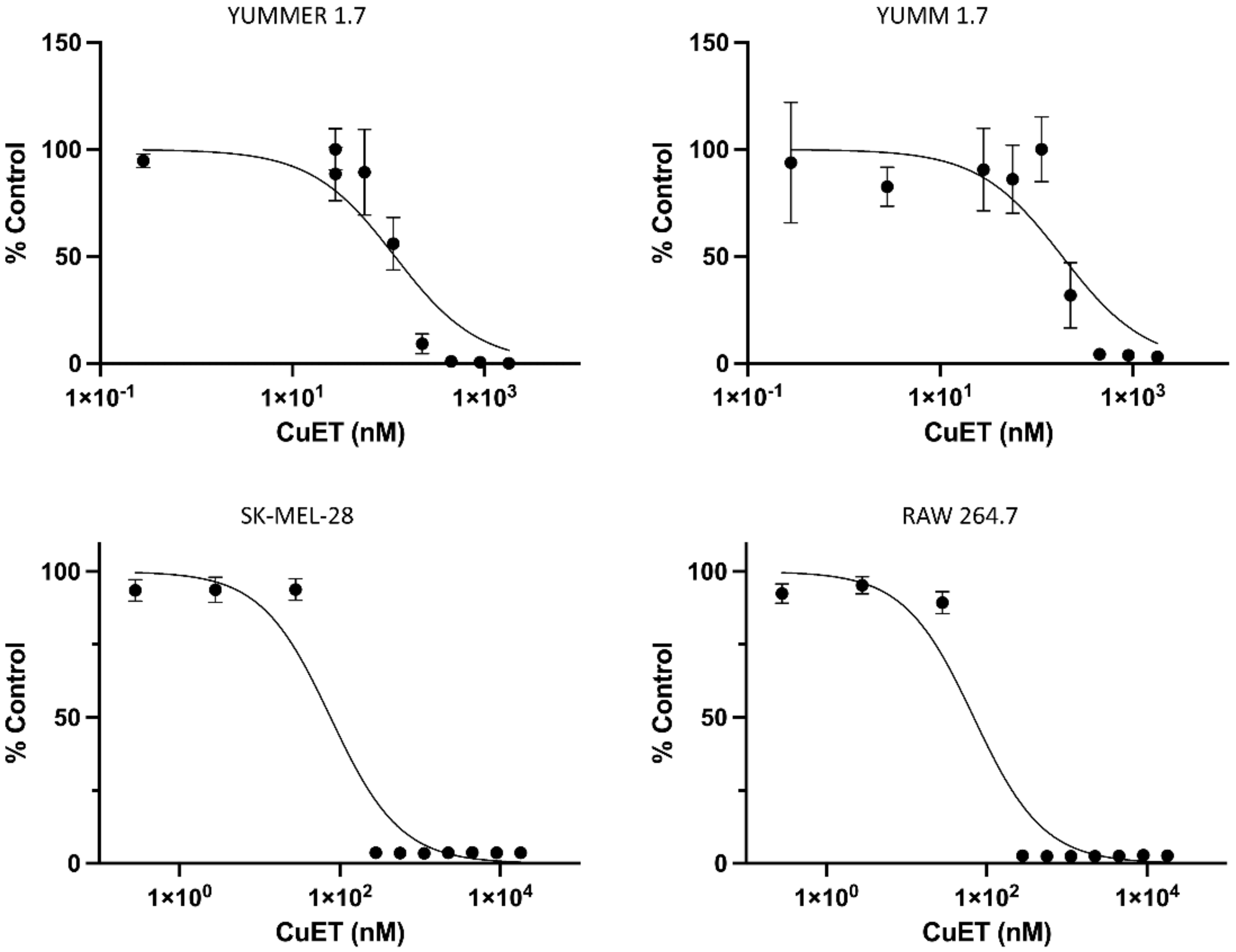

Figure S5. Representative IC50 curves of individual experiments of LP-CuET treated cells. IC50 values were obtained by taking the CuET concentration at 50%. Data plotted as mean ± SD, n = 3.

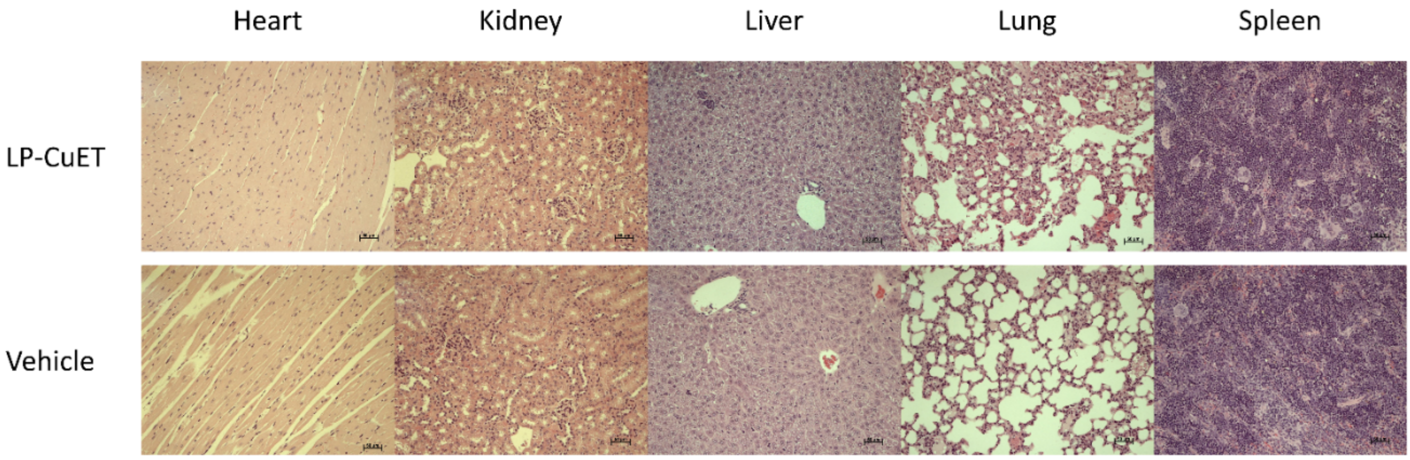

Figure S6. Hematoxylin and eosin (H&E) staining of major mouse organs. After 24 h of 1 mg.kg^{-1} LP-CuET treatment, mice were sacrificed and organs excised, fixed in 10% formalin, stained and imaged. No major morphological sign of acute cytotoxicity or tissue necrosis is observed in any of the studied organs. Scale bar: 50 μm.
